# MicroRNA-200 Family Modulation in Distinct Breast Cancer Phenotypes

**DOI:** 10.1371/journal.pone.0047709

**Published:** 2012-10-24

**Authors:** María Ángeles Castilla, Juan Díaz-Martín, David Sarrió, Laura Romero-Pérez, María Ángeles López-García, Begoña Vieites, Michele Biscuola, Susana Ramiro-Fuentes, Clare M. Isacke, José Palacios

**Affiliations:** 1 Instituto de Biomedicina de Sevilla-CSIC-Universidad de Sevilla, Hospital Universitario Virgen del Rocío, Department of Pathology, Seville, Spain; 2 Red temática de investigación cooperativa en cáncer (RTICC), Spain; 3 Breakthrough Breast Cancer Research Centre, The Institute of Cancer Research, London, United Kingdom; 4 Hospital Universitario Ramón y Cajal, Department of Pathology, Madrid, Spain; University of Edinburgh, United Kingdom

## Abstract

The epithelial to mesenchymal transition (EMT) contributes to tumor invasion and metastasis in a variety of cancer types. In human breast cancer, gene expression studies have determined that basal-B/claudin-low and metaplastic cancers exhibit EMT-related characteristics, but the molecular mechanisms underlying this observation are unknown. As the family of miR-200 microRNAs has been shown to regulate EMT in normal tissues and cancer, here we evaluated whether the expression of the miR-200 family (miR-200f) and their epigenetic state correlate with EMT features in human breast carcinomas. We analyzed by qRT-PCR the expression of miR-200f members and various EMT-transcriptional inducers in a series of 70 breast cancers comprising an array of phenotypic subtypes: estrogen receptor positive (ER+), HER2 positive (HER2+), and triple negative (TN), including a subset of metaplastic breast carcinomas (MBCs) with sarcomatous (homologous or heterologous) differentiation. No MBCs with squamous differentiation were included. The DNA methylation status of *miR-200f loci* in tumor samples were inspected using Sequenom MassArray® MALDI-TOF platform. We also used two non-tumorigenic breast basal cell lines that spontaneously undergo EMT to study the modulation of miR-200f expression during EMT in vitro. We demonstrate that miR-200f is strongly decreased in MBCs compared with other cancer types. TN and HER2+ breast cancers also exhibited lower miR-200f expression than ER+ tumors. Significantly, the decreased miR-200f expression found in MBCs is accompanied by an increase in the expression levels of EMT-transcriptional inducers, and hypermethylation of the *miR-200c-141 locus*. Similar to tumor samples, we demonstrated that downregulation of miR-200f and hypermethylation of the *miR-200c-141 locus,* together with upregulation of EMT-transcriptional inducers also occur in an *in vitro* cellular model of spontaneous EMT. Thus, the expression and methylation status of miR-200f could be used as hypothetical biomarkers to assess the occurrence of EMT in breast cancer.

## Introduction

Breast cancer constitutes a heterogeneous disease encompassing a large variety of entities with different clinical features. During the last decade gene expression profiling studies have established a molecular taxonomy of breast cancer defining four intrinsic molecular groups with clinical relevance: luminal A, luminal B, HER2+, and basal-like [1], [2], [3]. Luminal subtypes are defined by a gene expression signature partially resembling that of the luminal epithelial layer of the mammary gland, with positive expression of estrogen (ER) and/or progesterone (PR) receptors. HER2 tumors exhibit overexpression of the HER2 receptor and neighboring genes on 17q12–21 amplicon. Basal-like tumors are defined by the expression of basal/myoepithelial markers and generally lack the expression of ER, PR and HER2. Importantly, this molecular classification predicts treatment response and prognosis: Tumors classified into the basal-like and HER2 groups exhibit more aggressive clinical behavior compared to carcinomas with luminal gene expression signatures (reviewed in [4]).

**Figure 1:**
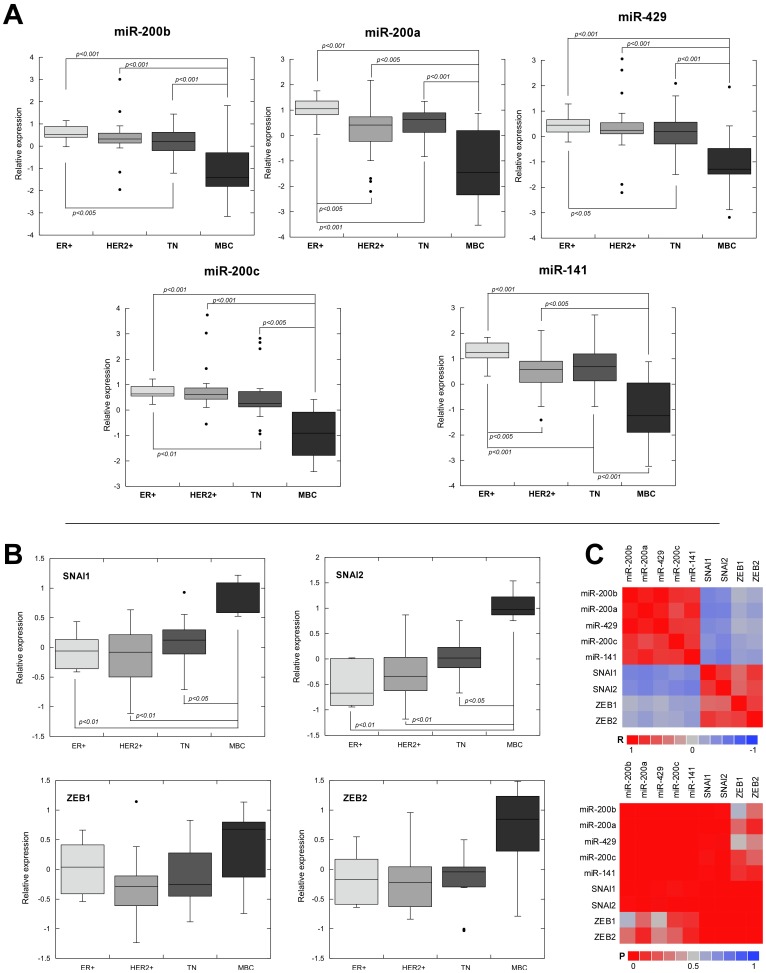
Expression profile of miR-200f and EMT-transcriptional inducers in breast tumors. The expression levels of miR-200 family members (**A**) and EMT-transcriptional inducers (**B**) were quantified by qRT-PCR in 70 breast cancer samples. Data are depicted as box-and-whisker plots. Adjusted *p*-values are shown where significant differences were found (Wilcoxon rank-sum test). ER+ (estrogen receptor positive tumors), HER2+ (HER2-positive tumors), TN (triple negative; ER−, PR−, HER2−), MBC (metaplastic breast carcinomas). (**C**) Upper heatmap represents Pearson coefficient (R) correlation values between the expression of EMT-transcriptional inducers and miR200f members. Bottom heatmap depicts the level of statistical significance (P) of the correlations.

Transcriptomic studies of breast cancer cell lines have corroborated this molecular classification and identified two clusters within the basal group: basal A, exhibiting basal/epithelial features, and basal B, composed by a subset of mesenchymal-like cell lines with enhanced invasive properties [5], [6], [7]. Similarly, recent studies in human breast cancers described a novel molecular subset of tumors within the basal-like phenotype, named claudin-low [8]. These claudin-low tumors resemble basal B breast cancer cell lines and are characterized by the enrichment for stem cell-like features and gene expression changes associated to EMT [8], [9], [10], [11].

EMT and the reverse process MET (mesenchymal-epithelial transition) are fundamental processes in developmental morphogenesis and have been proposed to play a key role in cancer development [12], [13]. Epithelial cells undergoing EMT lose cell-cell interactions and other epithelial traits while acquiring a migratory and invasive phenotype. This has led to the notion that EMT is an important event for tumor invasion and metastasis. Moreover, EMT appears to be associated with a stem cell-like phenotype that would endow tumor cells with the ability to self-renew, a necessary property to initiate colonization at distant metastasis [5], [13], [14], [15]. However, the precise occurrence of EMT in human cancer is still a matter of intense debate [16], due to the transient nature of the process and the possibility that it might only occur in specific tumor areas where cancer cells invade. In human breast cancer, we have previously reported that basal-like tumors and metaplastic breast carcinomas (MBCs) might undergo EMT-like processes [17]. MBCs, a specific subset of triple-negative tumors (TN, HER2–/ER–/PR–), are characterized by the presence of adenocarcinoma and metaplastic elements with squamous, sarcomatous or heterologous differentiation, and a clonal origin of both components has been suggested [18]. Therefore, MBCs represent extreme examples of phenotypic plasticity that may be related to the high aggressiveness and the characteristic metastatic spreading of these tumors. Indeed, MBCs, originally classified as basal-like tumors [19], show a strong claudin-low/EMT-enriched phenotype [17], [20].

**Figure 2:**
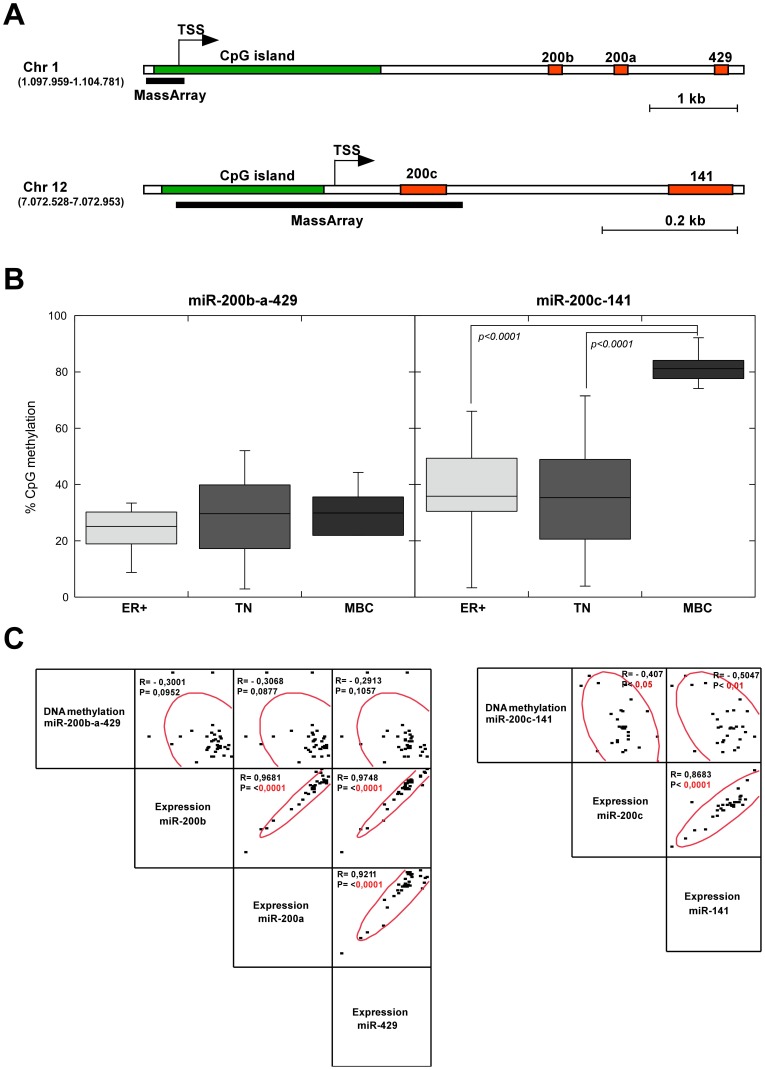
Analysis of DNA methylation status of miR-200f promoter sequences in breast tumors. (**A**) Schematic depiction of miR-*200b-a-429* and *miR-200c-141* genomic *loci* showing CpG islands (green), putative transcription start sites (TSS) and miRNA stem-loop sequences (red). The regions analyzed for DNA methylation (MassArray) are indicated by a black bar. Chromosomal location is indicated between brackets. (**B**) The DNA methylation levels across the regions shown in panel (**A**) were quantified in breast cancers by Sequenom MassArray® MALDI-TOF platform. The mean percentage of methylation levels in three tumor types (ER+, TN, MBC) are represented as box-plots. Statistical significance was determined by Student’s *t* test. (**C**) Expression and %CpG methylation data are represented on a scatter plot matrix showing correlation between the indicated variables, a 95% bivariate normal density ellipse is imposed on each scatterplot. Pearson correlation coefficients (R) and significance probabilities (P) are shown. Statistically significant *p* values (<0.05) are highlighted in red.

Functional E-cadherin loss is a hallmark of EMT. Several E-cadherin repressors, such as *SNAI1, SNAI2, ZEB1* and *ZEB2*, function as EMT-transcriptional inducers [21] and their overexpression in cancer cells associate to EMT features and generally aggressive tumors [22]. However, these transcription repressors are not the only EMT drivers. In the past few years several studies reported on the involvement of microRNAs (miRNAs) in controlling EMT [23], [24], [25]. miRNAs are small non-coding RNAs (∼23 nt) that silence gene expression by pairing to the 3′UTR of target mRNAs to direct their posttranscriptional repression [26]. Members of the miR-200 family (miR-200f) have been shown to be major regulators of EMT through the targeted silencing of the EMT-transcriptional inducers *ZEB1* and *ZEB2*, which in turn transcriptionally repress miR-200f in a double-negative feedback loop [27], [28]. In addition, miR-200f opposes EMT by directly targeting genes involved in motility and invasion [29], [30]. Therefore, expression of miR-200f in normal and cancer cells promotes the maintenance of an epithelial phenotype [31]. The five members of the miR-200f are grouped in two independent transcriptional units: a) *miR-200b*, *miR-200a* and *miR-429* are clustered in an intergenic region of chromosome 1; and b) *miR-200c* and *miR-141* are located on chromosome 12, within the intron of a noncoding RNA. Recent studies have shown that both clusters are subject to epigenetic regulation in cancer and normal tissue [32], [33], [34], [35]. Indeed, epigenetic regulation appears to be a dynamic process affecting miR-200f expression to modulate EMT-MET [36].

**Figure 3:**
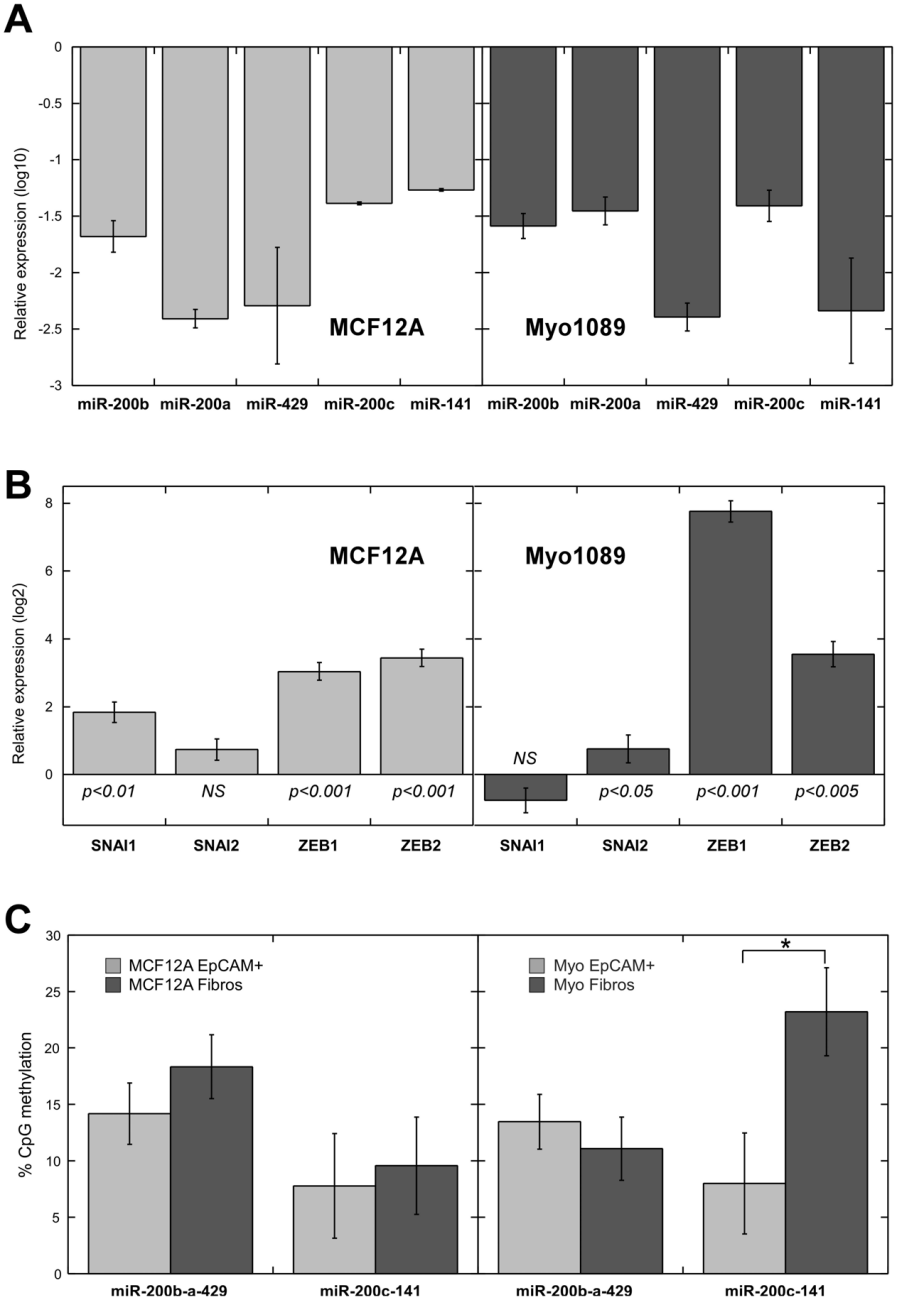
miR-200f and EMT-transcriptional inducers are modulated during *in vitro* EMT in breast basal cell lines. Expression of (**A**) miR-200f and (**B**) EMT-transcriptional inducers was evaluated by qRT-PCR in the sorted epithelial (EpCAM+) and mesenchymal (Fibros) subpopulations within MCF12A and Myo1089 cell lines. Data are normalized to the expression of *RNU48* and *18S* respectively. Bars represent mean expression changes ±SE in Fibros subpopulation relative to EpCAM+ cells (baseline). Three biological replicates were measured. Moderated t-test was performed to evaluate statistical significance (*p*<0.001 for all miRNAs analyzed in panel A. *NS*, non significant). (**C**) Methylation status of *miR-200f* promoter sequences in EpCAM+ and Fibros subpopulations within MCF12A and Myo1089 cell lines. Histogram bars represent averaged methylation levels (±SE) for the promoter sequences of *miR-200f loci*. Statistically significant differences are indicated (*p<0.001, Student’s t-test).

Since miR-200f is an important modulator of EMT, a comprehensive study on the expression of miR-200f members in the broad spectrum of breast cancer tumor types is warranted. Here we have analyzed the expression pattern of miR-200f members and their DNA methylation status in a series of breast tumors comprising ER+, HER2+, TN, and MBC types, as well as in a panel of breast cancer cell lines, and an *in vitro* model of spontaneous EMT.

## Methods

### Ethics Statement

This study was performed following standard ethical procedures of the Spanish regulation (Ley de Investigación Orgánica Biomédica, 14 July 2007) and was approved by the ethics committee of the Hospital Virgen del Rocío de Sevilla and the Fundación Pública Andaluza para la Gestión de la Investigación en Salud de Sevilla (FISEVI), Spain. Written informed consent has been obtained and all clinical investigation has been conducted according to the principles expressed in the Declaration of Helsinki.

### Human Tumors

A series of 70 formalin-fixed paraffin-embedded (FFPE) human breast tumors were obtained from the archives of the Department of Pathology of the Hospital Universitario Virgen del Rocío, Sevilla, Spain. The series included 16 luminal breast carcinomas (ER+/PR+/HER2−), 21 HER2 carcinomas (HER2+/ER−/PR−), 26 TN tumors (HER2−/ER−/PR−), and 7 MBCs (which are triple negative as well). Clinical data of the tumor sample are provided in **Table S1**. Carefully examination of the H&E-stained sections was carried out by pathologists (JP, MAL, BV) confirming the diagnosis on the basis of histological criteria. Immunohistochemistry was carried out on tissue microarray sections using the Envision method (Dako, CA, USA) and primary antibodies for E-cadherin (Zymed, CA, US) and Vimentin (Novocastra, UK). Immunohistochemical staining was evaluated by pathologists (JP, MAL, BV).

### Cell Lines

Six breast cancer cell lines with different phenotypes - MCF7, T47D, BT-474, SKBR3, MDA-MB-231 and BT549 - were obtained from the American Tissue Culture Collection (ATCC) and cultured according to ATCC’s recommendation. The non-tumorigenic basal cell lines MCF12A (from ATCC) and Myo1089 [37](kindly provided by Dr Michael Allen and Dr Louise Jones, Queen Mary’s University London) were grown in DMEM:F12 medium supplemented with 5% horse serum (Invitrogen, Life Technologies, Paisley, UK), 20 ng/ml EGF (PreproTech, London, UK), 500 ng/ml hydrocortisone (Sigma, Poole, UK), 100 ng/ml cholera toxin (Sigma), and 10 µg/ml insulin (Sigma). These non-tumorigenic basal cell lines were utilized as models for spontaneous EMT as previously reported [38]. The epithelial and mesenchymal-like subpopulations within these cell lines were purified by FACS sorting using EpCAM and CD49f surface markers as described before [38].

### Nucleic Acid Isolation

Total RNA was extracted in triplicate from 90% confluent cells using mirVana miRNA isolation kit (Ambion, Austin, TX, USA). For FFPE tumors samples, RNA was isolated from selected areas containing >70% tumor cells. After deparaffinization, the tissue was homogenized for 2 minutes in lysis buffer, using an Ultra-turrax (T10 Basic, IKA, Germany) according to the manufacturer’s protocol (Recover All™ total nucleic acid isolation kit, Ambion). DNA from FFPE tumors samples and cell lines were prepared using QIAGEN QIAamp ™ DNA FFPE Tissue Kit and QIAamp® DNA Mini Kit respectively, according to the manufacturer’s instructions (QIAGEN). The quantity and quality of RNA and DNA were measured with the NanoDrop ND-100 Spectrophotometer (Thermo Scientific, USA).

### Real-Time qRT-PCR

miRNA expression was measured by qRT-PCR using customized Taqman miRNA Arrays (Applied Biosystems). In brief, 700 ng total RNA was subjected to reverse transcription using the TaqMan MicroRNA Reverse Transcription Kit (Applied Biosystems) according to the manufacturer’s instructions. Megaplex™ RT primers (Human Pool A) were used for reverse transcription and thermal-cycling was performed over 40 cycles (16°C, 2 min; 42°C 1 min; 50°C 1 min). qRT-PCR reactions were performed on an Applied Biosystems 7900HT Fast Real Time PCR System (95°C 10 min, 40 cycles of 95°C 15 sec and 60°C 60 sec). *RNU48* was selected as endogenous control for normalization due to its consistent expression among all samples.

For gene expression analysis we used 800 ng of total RNA for the reverse transcription using the High Capacity RNA to cDNA kit (Applied Biosystems) according to the manufacturer’s protocol. qRT-PCR reactions were performed using Taqman Array Cards (Applied Biosystems) including probes for: *SNAI1, SNAI2, ZEB1* and *ZEB2*. *18S* was used as endogenous control to normalize variations in the quantities of input cDNA. Applied Biosystems 7900HT Fast Real Time PCR System was used with the following PCR conditions: 50°C 2 min; 94.5°C 10 min; 40 cycles of melting 97°C 30 sec and annealing 59.7°C 1 min.

Relative mRNA and miRNA expression levels were calculated by the formula 2^−ΔΔCt^ using SDS software (Applied Biosystems).

### DNA Methylation Analysis

1 µg of genomic DNA was used for sodium bisulfite conversion using EpiTect® Plus DNA Bisulfite kit (QIAGEN) following the supplied protocols. Primers for PCR amplification of the converted DNA were designed using EpiDesigner® software (Sequenom, San Diego, CA, USA). Two overlapping amplicons were analyzed in the promoter region of each genomic *locus* (*miR-200b-a-429* and *miR-200c-141*). Primer sequences are shown in **Table S2**. Amplicons were analyzed using the MassARRAY MALDI-TOF mass spectrometry platform (Sequenom). Data were analyzed using EpiTyper software (Sequenom) and the methylation level of the promoter sequences was calculated by averaging the methylation level of the individual CpG units.

### Statistical Analysis

Statistical significance of relative changes in miRNA and mRNA expression between different groups of breast tumors was determined by the Wilcoxon rank-sum test, correcting p-values with the Benjamini-Hochberg algorithm (p<0.05). A moderate t-test was used when analyzing statistical differences in cell line expression data. These analyses were conducted on Integromics RealTime StatMiner™ 4.2 package (Integromics S.L., Granada, Spain). SPSS (SPSS inc., Chicago, IL) and JMP statistical software (SAS Institute Inc., Cary, NC, US) were used to perform the correlation analyses. Correlation of the normalized expression data and %CpG methylation data was determined using the Pearson coefficient.

## Results and Discussion

### miR-200f Expression Decreases in Breast Tumors with EMT Features

We investigated miR-200f expression pattern in 70 human breast cancers comprising ER+, HER2+, and TN tumor subtypes, including a subset of MBCs with sarcomatous (homologous or heterologous) differentiation (**Figure 1A**). No MBCs with squamous differentiation were included. We found a significant decrease of all miR-200f members in MBCs compared to the rest of tumor types. Consistent with this, basal B breast cancer cell lines, which have been proposed to represent *in vitro* models of MBC and claudin-low tumors [5], exhibited significantly reduced levels of miR-200f expression compared to luminal cell lines (**Figure S1**). Moreover, compared with ER+ breast tumors, TN cancers also exhibited decreased expression of the whole mir-200f, while HER2+ cancers only showed significant downregulation of miR-200a and miR-141 (**Figure 1A**). Hence, miR-200f expression was higher in ER+ tumors, whereas TN and HER2+ tumors exhibited intermediate levels, and MBCs showed strong reduction of miR-200f compared with the rest of groups. Reduced levels of miR-200f in MBCs and claudin-low tumors relative to other tumor phenotypes has been previously reported, but detailed analysis of miR-200f in a variety of human breast cancer phenotypes were not carried out in these studies [39], [40]. Bockmeyer et al [41] analyzed miRNA expression in a profiling experiment comparing luminal A, luminal B, basal-like and malignant myoepithelioma, and observed low level miR-200c and mir-429 expression restricted to malignant myoepithelioma. However, our larger series of distinct breast cancer phenotypes allowed us to detect a significant reduction in all miR-200f members in TN tumors, in addition to the marked reduction observed in MBCs. As expected, by immunohistochemical analysis we observed that TN and MBC tumors were positively associated to the mesenchymal marker vimentin, and ER+ tumors were associated to the epithelial marker E-cadherin (**Figure S2**). Therefore, given that basal-like tumors are suggested to be particularly prone to acquire EMT-like characteristics [17] and MBCs are considered as examples of complete EMT [9], [10], [11], our data indicate that the expression of miR-200f could be an ideal biomarker to assess the degree of occurrence of EMT in breast cancer.

In order to assess further the association between miR-200f and EMT transcriptional regulation in breast cancer we analyzed the expression of diverse EMT-transcriptional inducers including *ZEB1* and *ZEB2*, which have been reported to directly inhibit transcriptional activation of miR-200f expression through conserved E-box elements [27], [28]. We found a strong and significant increase in the expression of *SNAI1* and *SNAI2* and a moderate but not statistically significant increase of *ZEB1* and *ZEB2* in MBCs compared with the rest of tumor types (**Figure 1B**). There were no additional differences in the rest of tumor groups for any of the analyzed genes. In agreement with our observations in tumor samples, we detected higher levels of EMT-transcriptional inducers in basal B cell lines, which exhibited upregulation of *SNAI2*, *ZEB1* and *ZEB2* (**Figure S1**). These results indicate that EMT-transcriptional inducers could account for mir-200f downregulation in our tumor series. Accordingly, we observed significant negative correlations between *SNAI1/SNAI2* (but not *ZEB1* or *ZEB2*) and all miR-200f members’ expression levels in our tumor series (**Figure 1C** and **Figure S3**). Since *SNAI1/SNAI2* are *E-cadherin* repressors affecting promoter transcriptional activity through binding to E-box elements, it is conceivable that these EMT-transcriptional inducers could affect transcriptional activation of miR-200f through the conserved E-box elements that have been functionally defined in previous studies [27], [42]. Consistent with this idea, we demonstrated that overexpression of *SNAI2* in MDCK cells had a strong repressive effect in the transcriptional activation of *miR-200b-a-429* promoter, and this effect was strictly dependent on E-box motifs (**Figure S4**). This observation supports the notion that other EMT-transcriptional inducers, distinct from *ZEB1* and *ZEB2*, could directly regulate miR-200f expression. In fact, during the preparation of this manuscript a study has been published describing a mutually inhibitory regulatory loop for *SNAI2* and miR-200b/miR-1 in a murine model of aggressive prostate cancer. Transcriptional repression of *miR-200b-a-429* and *miR-1* by *SNAI2* occurs by direct interaction through E-box elements; and conversely, *SNAI2* was functionally validated as target gene for *miR-1* and *miR-200b* [43]. As *miR-1* and *miR-200* target sequences identified in *SNAI2* 3′-UTR are well conserved, mutually repressive regulation for *SNAI2* and miR-1/miR-200b-a-429 is likely to be a common regulatory mechanism operating in human and other species. *SNAI2* has been described as the EMT-transcriptional inducer responsible for conferring a basal-like phenotype in breast cancer [44], [45], so it would be possible that this new regulatory axis plays an important contribution to the EMT-like features observed in these tumors.

### Hypermethylation of miR-200c-141 Promoter Sequences in MBCs

We next studied the possible mechanisms by which miR-200f expression could be regulated in the different breast cancer subtypes. Previous studies have shown that mir-200f is subjected to epigenetic regulation in cancer and normal tissues [32], [33], [34], [35], [36], [42]. The promoter sequences driving transcriptional activation of both miR-200f clusters are located within CpG islands, and DNA methylation in these CpG islands is associated to inactivation of miR-200f in cancer [46]. We therefore evaluated the CpG methylation rates of the respective miR-200f promoter regions in our tumor series using the Sequenom MassArray® MALDI-TOF platform. The promoter sequences analyzed for *miR-200b-a-429* cluster span 415 bp encompassing the transcription start site (TSS) functionally defined by Bracken and colleagues [27]. For *miR-200c-141* cluster we studied a region of 426 bp surrounding the TSS that has also been previously determined [42] (**Figure 2A**).

For these analyses we focused our study in ER+, TN and MBC tumors and observed that DNA methylation levels of *miR-200c-141 locus* were significantly higher in MBCs compared with either ER+ or TN tumors (**Figure 2B**). Although the methylation levels of *miR-200b-a-429 locus* were slightly increased in MBCs, there were no significant differences among the different tumor types. Interestingly, the expression of *miR-200c-141* and its DNA methylation levels showed a significant inverse correlation in breast tumors, but no such association was observed for *miR-200b-a-429* cluster (**Figure 2C**). In agreement with these data in breast tumors, basal B cancer cell lines also exhibited elevated DNA methylation rates at *miR-200c-141* promoter, reaching levels >80%, as observed in MBCs. Additionally, we also detected elevated methylation at *miR-200b-a-429 locus* in basal B cell lines (>50%), that correlates with downregulation of miR-200b, miR-200a and miR-429 (**Figures S5** and **S1**). Taken together, these results indicate that DNA methylation is a major epigenetic mechanism regulating transcriptional activation of *miR-200c-141* cluster in MBCs/basal B cancer cells and suggest that this epigenetic event is associated with the acquisition of EMT features in these tumors.

A recent study [47] has functionally defined an independent promoter region for *miR-200b-a-429* cluster, capable of directing transcriptional activity at the same level as the promoter sequences formerly described [27] and studied here. Remarkably, the two promoter sequences were differentially methylated in breast cancer clinical specimens suggesting different regulatory roles [47]. Similarly, Vrba et al [33] described that normal human mammary cells exhibit differential methylation of the proximal and distal parts of the CpG island (**Figure 2A**), where the respective *miR-200b-a-429* promoter regions are located. Wee et al [47] have also shown that methylation of both promoter regions is inversely associated with miR-200b expression levels in breast cancer cell lines as well as in clinical specimens. However, we did not observe such correlation in our tumor series for the analyzed promoter region (**Figure 2C**). This discrepancy could be due to differences in sample size. The authors also reported that hypermethylation of the newly identified promoter, but not of the formerly defined one, was associated with loss of ER+ or PR+ expression, and gain of HER2+ phenotype. In agreement with this we did not observe differences in DNA methylation levels of the former promoter region in TN or MBCs (which lack expression of ER and PR), compared with ER+ tumors (**Figure 2B**).

It has recently been reported that the two clusters comprising the miR-200f display different but complementary epigenetic mechanisms to regulate their expression in human mammary cells [33]. The authors describe that *miR-200b-a-429* cluster is epigenetically repressed in mammary fibroblasts by the repressive histone mark H3K27me3, while DNA methylation is the major epigenetic mechanism repressing the transcriptional activation of *miR-200c-141* cluster. Our results are in accordance with these observations, since we only observed expression/DNA methylation correlation for *miR-200c-141* cluster (**Figure 2C**). Nevertheless, it would be necessary to determine other epigenetic marks in order to establish the actual epigenetic state of *miR-200f loci*. Indeed, in hepatocellular carcinoma, *miR-200b-a-429* promoter sequences show decreased histone H3 acetylation, a permissive epigenetic mark that is in turn modulated by miR-200a through direct targeting of the histone deacetylase 4 (HDAC4) [48].

### Spontaneous EMT in Normal Basal Breast Cell Lines is Associated to Hypermethylation of miR-200c-141 Locus

To study further the association between modulation of miR-200f and induction of EMT in breast cancer and to validate the epigenetic regulation of miR200 during this process, we utilized our recently described model systems for spontaneous EMT in mammary cells [38]. We have demonstrated that the immortalized non-tumorigenic cell lines MCF12A and Myo1089 contain two functionally and phenotypically distinct subpopulations: EpCAM^pos^/CD49f^high^ cells (referred as to ‘EpCAM+’) show the typical cobblestone epithelial morphology and phenotypically resemble basal A breast cancers, while EpCAM^neg^/CD49f^med/low^ cells (‘Fibros’) show fibroblast-like spindle morphology and exhibit a gene expression pattern resembling stromal fibroblast and Basal-B/claudin-low cancers [38]. Importantly, the Fibros subpopulation arises from EpCAM+ cells through an EMT-like process.

Here, we sorted these two subpopulations from MCF12A and Myo1089 cells and studied the expression of the miR-200f members and their DNA methylation status as well as expression of EMT-transcriptional inducers. As expected, the expression levels of all of miR-200f members showed a significant decrease in Fibros compared to EpCAM+ subpopulation in both cell lines (**Figure 3A**). Moreover, downregulation of miR-200f in the Fibros subpopulation is accompanied by an increase in the expression levels of *ZEB1* and *ZEB2* (**Figure 3B** and [38]), while differential expression of *SNAI1* and *SNAI2* was only observed in MCF12A and Myo1089 cell lines, respectively. Importantly, Fibros subpopulation from Myo1089 cells showed increased methylation level of *miR-200c-141 locus* but not of *miR-200b-a-429 locus* (**Figure 3C**), which parallels the result observed in MBCs (**Figure 2B**). No differential DNA methylation levels were found in the MCF12A cell line, but this could be due to the fact that EMT in this cell line is more transient than in Myo1089 cells [38]. Overall, these data reproduced what we observed in MBCs, which exhibited strong reduction of miR-200f with hypermethylation at *miR-200c-141 locus*, and increased levels of EMT-transcriptional inducers.

### Conclusion

We have observed that TN and specially MBCs breast cancers exhibit lower level expression of miR-200f compared to ER+ tumors. Using an in vitro model of EMT and the analysis in human breast cancer samples we have shown that the strong decrease of miR-200f expression levels is accompanied by upregulation of EMT-transcriptional inducers and hypermethylation of *miR-200c-141 locus*, thus supporting the functional association between EMT and miR-200f regulation in breast cancer. Therefore, our data indicate that expression and methylation status of miR-200f could be used as a hypothetical biomarker to assess the occurrence of EMT in breast cancer both *in vitro* and *in vivo*.

## Supporting Information

Figure S1 **Expression levels of miR-200f and EMT-transcriptional inducers measured by qRT-PCR in breast cancer cell lines.** (**A**) Strong downregulation of miR-200f is observed in mesenchymal-like basal B cell lines (MDA-MB-231, BT-549) compared with luminal (MCF7, T47D) and HER2+ (SKBR3, BT474) cell lines. Expression levels are normalized to *RNU48*. (**B**) Basal B cell lines (MDA-MB-231, BT-549) exhibit higher expression of *SNAI2*, *ZEB1* and *ZEB2* genes than luminal and HER2+ breast cancer cells. Expression levels are normalized to *18S*.
(PDF)

Figure S2 **Immunohistochemistry** **for E-cadherin and Vimentin.** (**A**) Representative IHC pictures for E-cadherin and Vimentin expression in ER+ and MBC tumors. (**B**) Scoring data for IHC are expressed as percentage. The scoring criteria for immunohistochemical staining according to the threshold of positive staining for each marker was as follows: E-cadherin, absent : no expression, reduced: less than 50% of epithelial cells with complete and intense membrane expression, conserved 50% or more epithelial cells with complete and intense membrane expression; vimentin positive >10% of positive epithelial cells.
(PDF)

Figure S3 **Correlations on expression levels of miR-200f members and EMT-transcriptional inducers in breast tumors.** Data shown are an extension from those in Figure 1. Pearson correlation coefficients (R) and significance probabilities (P) for each gene pair are shown. Darker shading indicates significant correlations.
(PDF)

Figure S4 **Stable overexpression of** ***SNAI2*** **represses transcriptional activation of** ***miR-200b-a-429*** **promoter through E-box elements.** MDCK-CMV (control) and MDCK-SNAI2 cell lines were transfected with firefly luciferase reporter constructs containing the promoter sequences of mouse *E-cadherin* (−178/+92) or human *miR-200b-a-429* (−321/+120). Constructs containing the WT promoter sequences or mutated E-box elements were utilized. Relative luciferase activity of the WT constructs was set to 1. A strong de-repressive effect was observed for miR-200b-a-429 E1E2 mutant promoter in the cell line overexpressing *SNAI2*. Bars represent mean fold change in relative luciferase activity ± SE in three independent experiments.
(PDF)

Figure S5 **DNA methylation** ***status*** **of** ***miR-200f loci*** **in breast cancer cell lines.** Basal B cell lines (MDA-MB-231, BT-549) show higher methylation rates than luminal (MCF7, T47D) and HER2+ (SKBR3, BT474) cell lines in both *miR-200f loci*.
(PDF)

Table S1 **Clinical characteristics of the tumor series.**
(PDF)

Table S2 **Primers used for DNA methylation analysis.**
(PDF)
