# Supplementary figures and images for: MicroRNA-200 Family Modulation in Distinct Breast Cancer Phenotypes
Source: PLoS One. 2012 Oct 24;7(10):e47709. doi: 10.1371/journal.pone.0047709 (PMC3480416; doi:10.1371/journal.pone.0047709)

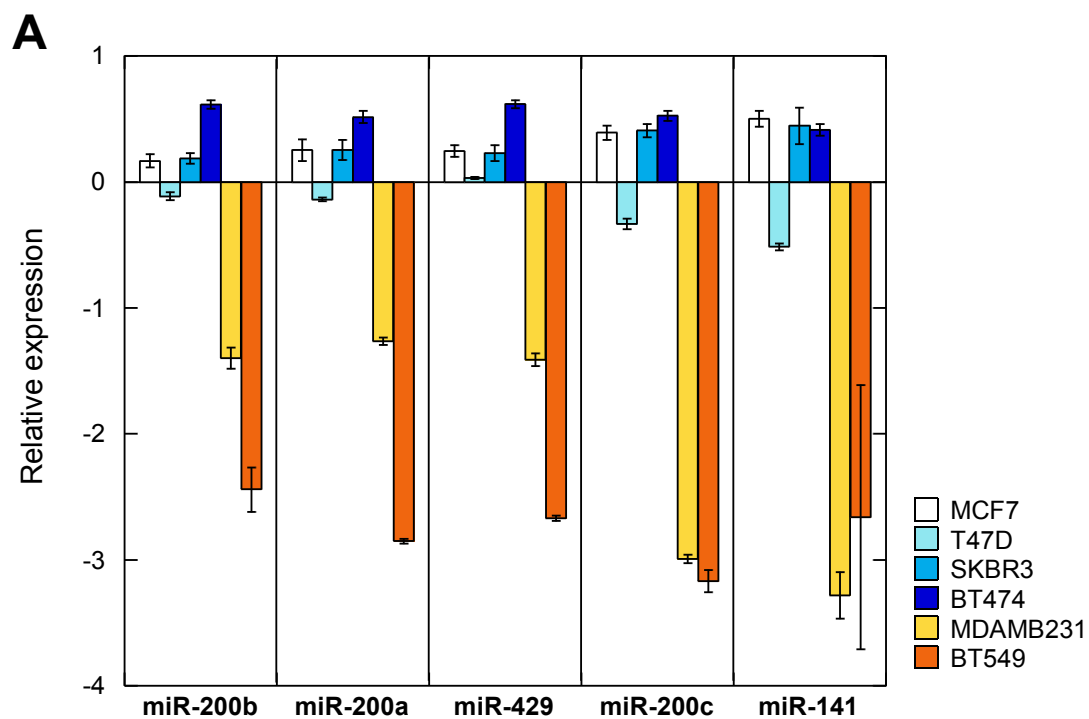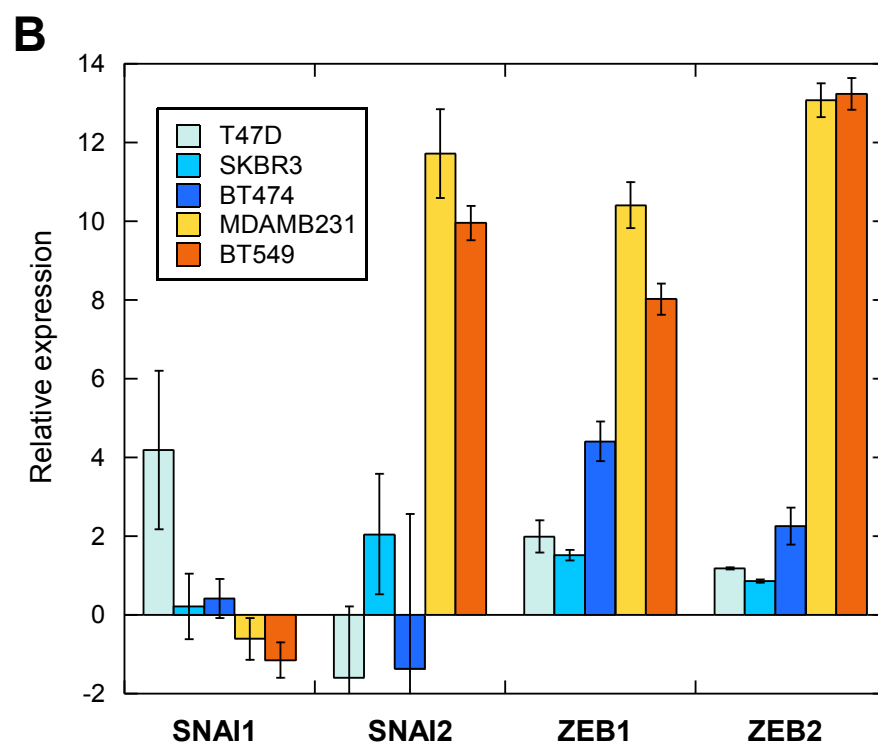

Supplement: Figure S1 — Expression levels of miR-200f and EMT-transcriptional inducers measured by qRT-PCR in breast cancer cell lines. (A) Strong downregulation of miR-200f is observed in mesenchymal-like basal B cell lines (MDA-MB-231, BT-549) compared with luminal (MCF7, T47D) and HER2+ (SKBR3, BT474) cell lines. Expression levels are normalized to RNU48. (B) Basal B cell lines (MDA-MB-231, BT-549) exhibit higher expression of SNAI2, ZEB1 and ZEB2 genes than luminal and HER2+ breast cancer cells. Expression levels are normalized to 18S. (PDF) [file pone.0047709.s001.pdf]

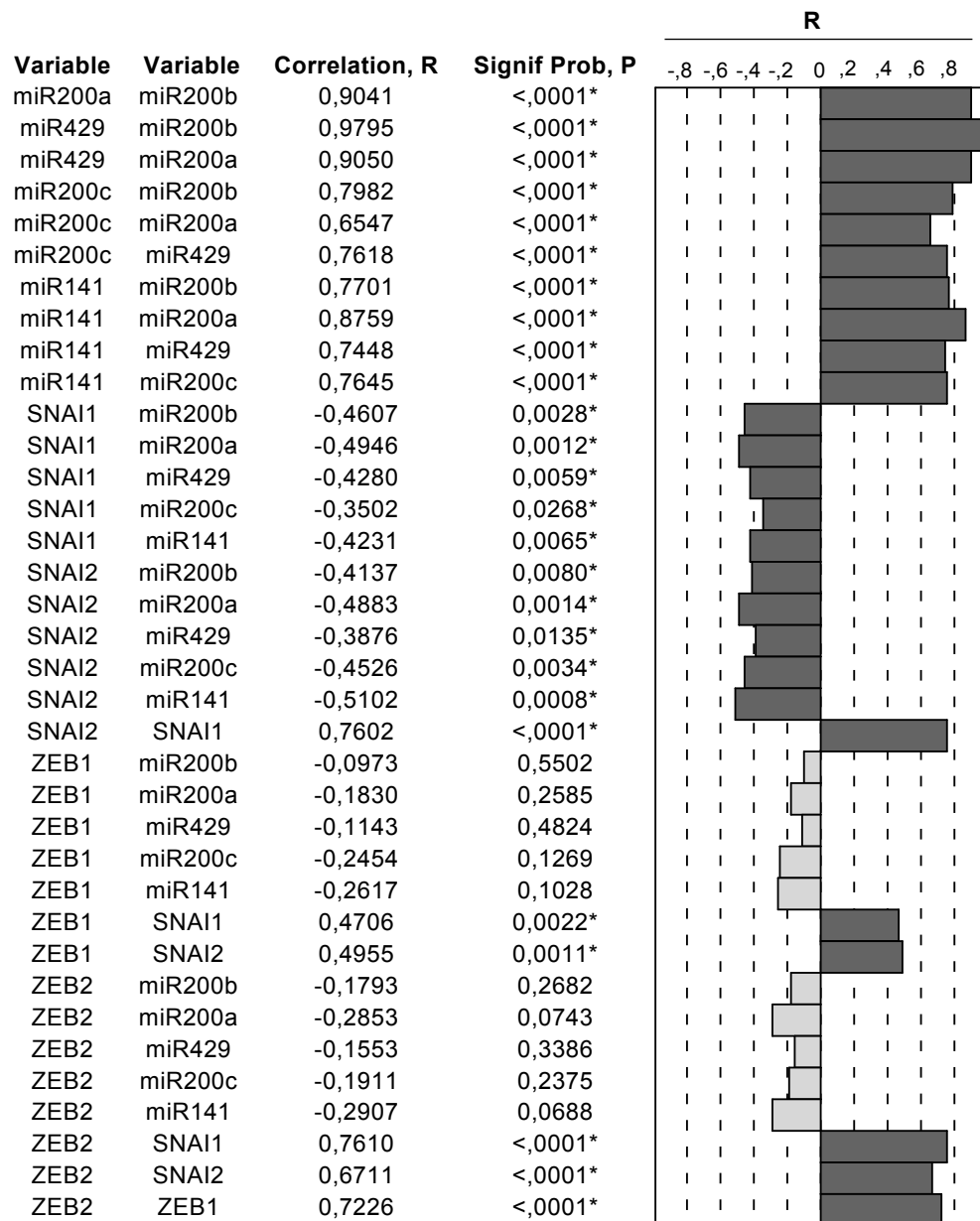

\* significance level <0.05

Supplement: Figure S3 — Correlations on expression levels of miR-200f members and EMT-transcriptional inducers in breast tumors. Data shown are an extension from those in Figure 1. Pearson correlation coefficients (R) and significance probabilities (P) for each gene pair are shown. Darker shading indicates significant correlations. (PDF) [file pone.0047709.s003.pdf]

# miR-200b-a-429 Promoter::LUC

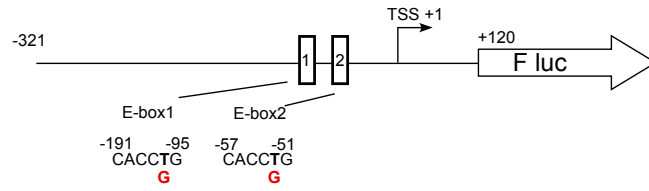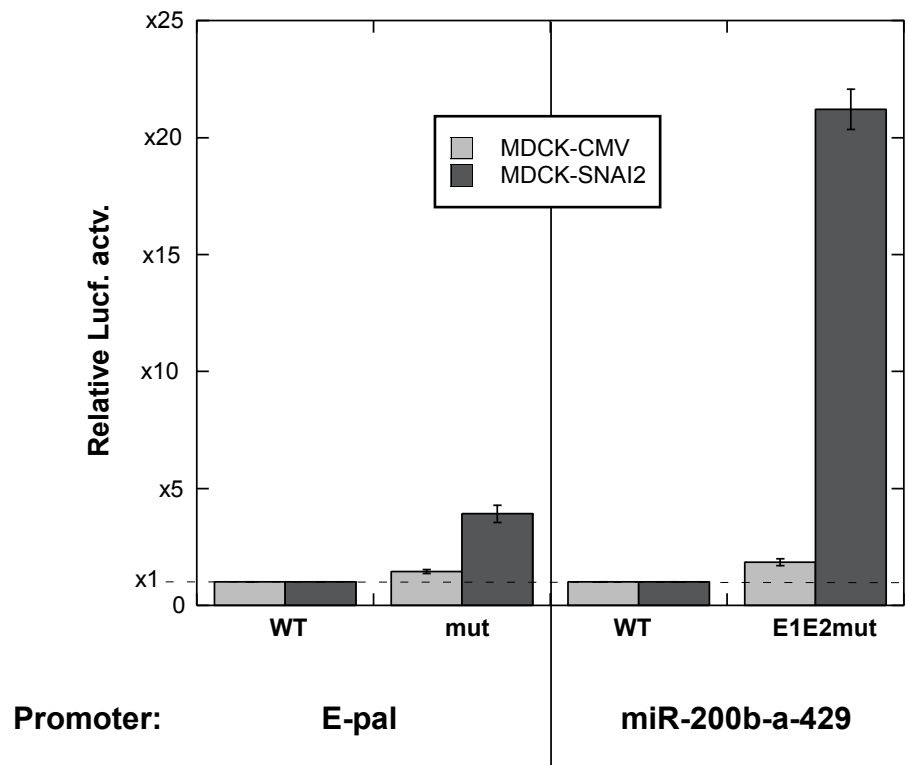

Supplement: Figure S4 — Stable overexpression of SNAI2 represses transcriptional activation of miR-200b-a-429 promoter through E-box elements. MDCK-CMV (control) and MDCK-SNAI2 cell lines were transfected with firefly luciferase reporter constructs containing the promoter sequences of mouse E-cadherin (−178/+92) or human miR-200b-a-429 (−321/+120). Constructs containing the WT promoter sequences or mutated E-box elements were utilized. Relative luciferase activity of the WT constructs was set to 1. A strong de-repressive effect was observed for miR-200b-a-429 E1E2 mutant promoter in the cell line overexpressing SNAI2. Bars represent mean fold change in relative luciferase activity ± SE in three independent experiments. (PDF) [file pone.0047709.s004.pdf]

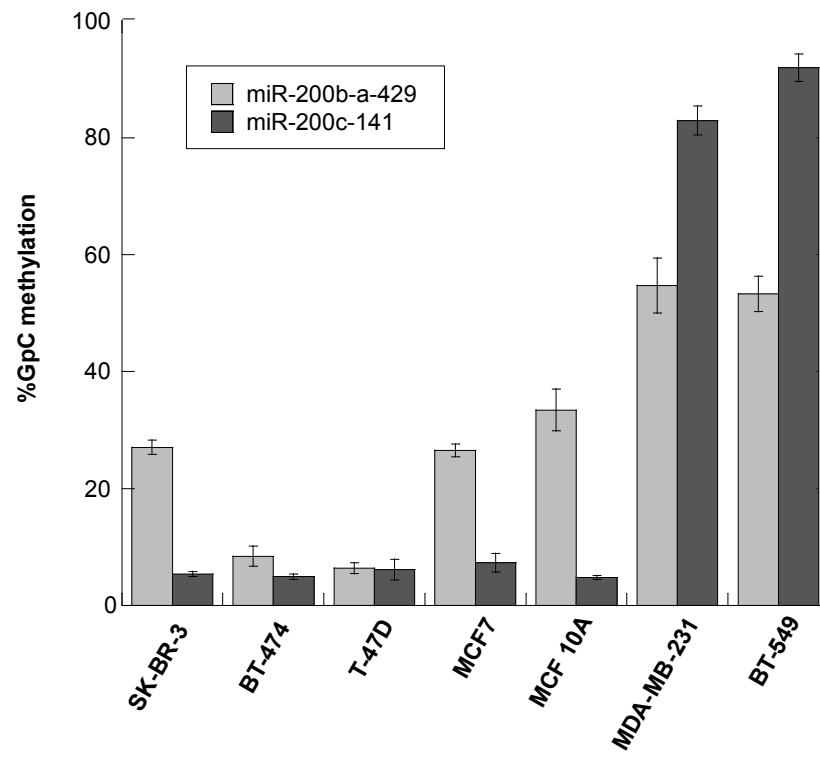

Supplement: Figure S5 — DNA methylation status of miR-200f loci in breast cancer cell lines. Basal B cell lines (MDA-MB-231, BT-549) show higher methylation rates than luminal (MCF7, T47D) and HER2+ (SKBR3, BT474) cell lines in both miR-200f loci. (PDF) [file pone.0047709.s005.pdf]
