# Supplementary material for: MicroRNA-200 Family Modulation in Distinct Breast Cancer Phenotypes
Source: PLoS One. 2012 Oct 24;7(10):e47709. doi: 10.1371/journal.pone.0047709 (PMC3480416; doi:10.1371/journal.pone.0047709)
Supplement: Figure S2 — Immunohistochemistry for E-cadherin and Vimentin. (A) Representative IHC pictures for E-cadherin and Vimentin expression in ER+ and MBC tumors. (B) Scoring data for IHC are expressed as percentage. The scoring criteria for immunohistochemical staining according to the threshold of positive staining for each marker was as follows: E-cadherin, absent : no expression, reduced: less than 50% of epithelial cells with complete and intense membrane expression, conserved 50% or more epithelial cells with complete and intense membrane expression; vimentin positive >10% of positive epithelial cells. (PDF) [file pone.0047709.s002.pdf]

A

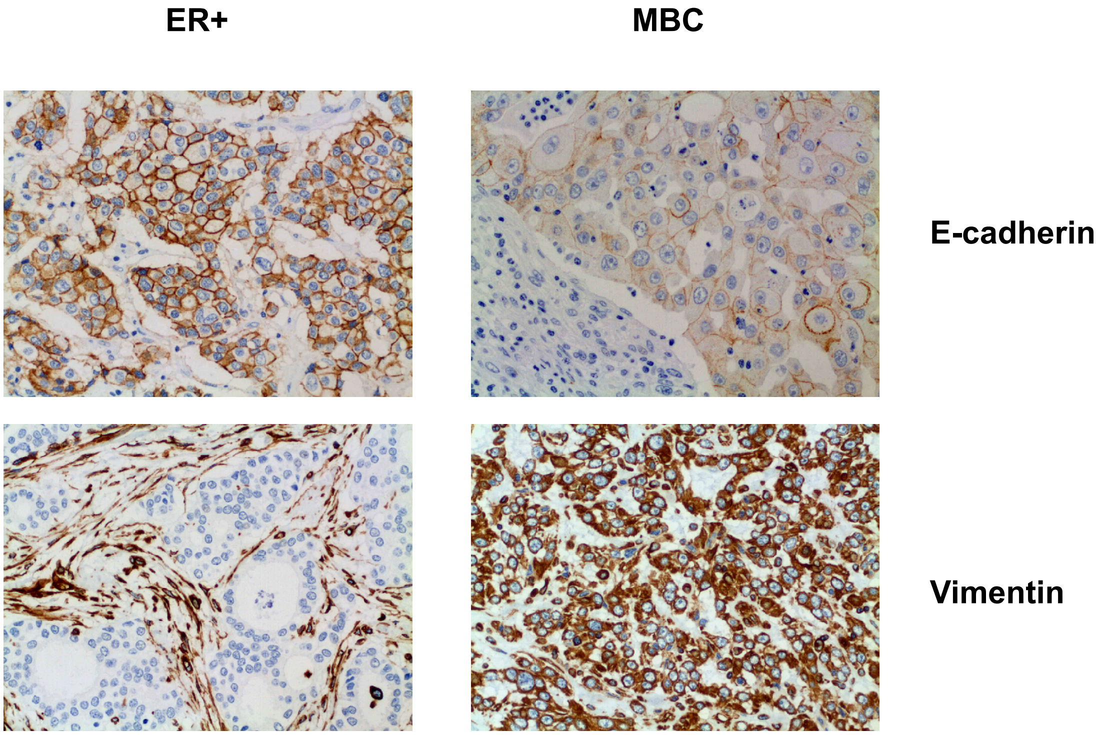

B

	E-cadherin			Vimentin	
Phenotype	Absent	Reduced	Conserved	Negative	Positive
ER+	6	51	43	89	11
TN	4	88	8	37	63
MBC	0	100	0	0	100
