# Supplementary material for: MicroRNA-200 Family Modulation in Distinct Breast Cancer Phenotypes
Source: PLoS One. 2012 Oct 24;7(10):e47709. doi: 10.1371/journal.pone.0047709 (PMC3480416; doi:10.1371/journal.pone.0047709)
Supplement: Table S1 — Clinical characteristics of the tumor series. (PDF) [file pone.0047709.s006.pdf]

Table S1: CLINICAL AND HISTOPATHOLOGICAL CHARACTERIZATION OF BREAST CARCINOMA

Patients	Clinical Phenotype	Histotype	AGE (years)	Tumor size (cm)	Histological grade	Stage	ER	PR	HER2	Ki-67 (%)
1	LUMINAL	Ductal	53	2.5	3	IIA	positive	positive	negative	20%
2	LUMINAL	Ductal	62	2.2	2	IIB	positive	positive	negative	10%
3	LUMINAL	Ductal	48	1.6	3	IA	positive	positive	negative	70%
4	LUMINAL	Ductal	52	0.9	1	IA	positive	positive	negative	10%
5	LUMINAL	Ductal	88	3.8	3	IIB	positive	positive	negative	10%
6	LUMINAL	Lobular	43	14	2	IIIC	positive	positive	negative	10%
7	LUMINAL	Ductal	48	2	2	IIIA	positive	positive	negative	10%
8	LUMINAL	Papillary	83	2.7	1	IIA	positive	positive	negative	10%
9	LUMINAL	Ductal	83	3	2	IIA	positive	positive	negative	10%
10	LUMINAL	Ductal	48	2.1	3	IIA	positive	positive	negative	10%
11	LUMINAL	Ductal	85	3	2	IIIA	positive	positive	negative	10%
12	LUMINAL	Ductal	82	2	2	IIA	positive	positive	negative	20%
13	LUMINAL	Mucinous	79	3.2	1	IIA	positive	positive	negative	20%
14	LUMINAL	Ductal	43	2.6	2	IIA	positive	positive	negative	10%
15	LUMINAL	Ductal	48	2.3	3	IIB	positive	positive	negative	10%
16	LUMINAL	Ductal	85	0.9	2	IA	positive	positive	negative	10%
17	HER2	Ductal	49	4	3	IIA	negative	negative	positive	20%
18	HER2	Ductal	54	5.5	2	IIIA	negative	negative	positive	50%
19	HER2	Ductal	56	3	3	IIA	negative	negative	positive	50%
20	HER2	Ductal	55	3.8	3	IIB	negative	negative	positive	70%
21	HER2	Ductal	45	2.4	3	IIIC	negative	negative	positive	60%
22	HER2	Ductal	49	2.8	3	IIIC	negative	negative	positive	30%
23	HER2	Ductal	65	1.5	3	IA	negative	negative	positive	10%
24	HER2	Ductal	65	2.5	3	IIIC	negative	negative	positive	30%
25	HER2	Ductal	76	2	3	IIIC	negative	negative	positive	30%
26	HER2	Ductal	53	5	3	IIIA	negative	negative	positive	20%
27	HER2	Ductal	31	1.8	3	IIIA	negative	negative	positive	20%
28	HER2	Ductal	51	1.8	3	IA	negative	negative	positive	30%
29	HER2	Ductal	40	12	3	IIIC	negative	negative	positive	20%
30	HER2	Ductal	59	0.8	3	IIA	negative	negative	positive	20%
31	HER2	Ductal	48	2.1	3	IIA	negative	negative	positive	20%
32	HER2	Ductal	49	3.5	3	IIB	negative	negative	positive	20%
33	HER2	Ductal	66	1.8	3	IIB	negative	negative	positive	20%
34	HER2	Ductal	63	0.8	2	IA	negative	negative	positive	20%

35	HER2	Ductal	55	2.7	3	IIIC	negative	negative	positive	20%
36	HER2	Ductal	64	3.2	3	IIIC	negative	negative	positive	40%
37	HER2	Ductal	36	3	3	IIB	negative	negative	positive	10%
38	Triple Negative	Apocrine	71	4.5	3	IIA	negative	negative	negative	20%
39	Triple Negative	Ductal	56	4	2	IIA	negative	negative	negative	10%
40	Triple Negative	Ductal	44	1.6	3	IA	negative	negative	negative	70%
41	Triple Negative	Ductal	49	4.5	2	IIA	negative	negative	negative	60%
42	Triple Negative	Ductal	61	5	3	IIIB	negative	negative	negative	40%
43	Triple Negative	Ductal	46	2.5	3	IIB	negative	negative	negative	30%
44	Triple Negative	Ductal	39	8	3	IIIC	negative	negative	negative	70%
45	Triple Negative	Medulary features	38	2.5	3	IIB	negative	negative	negative	50%
46	Triple Negative	Ductal	83	11	3	IIIB	negative	negative	negative	30%
47	Triple Negative	Ductal	91	7	3	IIB	negative	negative	negative	40%
48	Triple Negative	Ductal	40	1.9	3	IA	negative	negative	negative	70%
49	Triple Negative	Ductal	48	2.2	3	IIIA	negative	negative	negative	50%
50	Triple Negative	Ductal	38	4	3	IIB	negative	negative	negative	40%
51	Triple Negative	Ductal	63	2	3	IIA	negative	negative	negative	20%
52	Triple Negative	Ductal	53	2.2	3	IIA	negative	negative	negative	40%
53	Triple Negative	Ductal	62	2.5	3	IIB	negative	negative	negative	50%
54	Triple Negative	Ductal	38	2.5	3	IIB	negative	negative	negative	70%
55	Triple Negative	Ductal	77	4.2	3	IIIA	negative	negative	negative	70%
56	Triple Negative	Ductal	43	3	3	IIA	negative	negative	negative	50%
57	Triple Negative	Ductal	48	4	3	IIB	negative	negative	negative	40%
58	Triple Negative	Ductal	46	3.2	3	IIB	negative	negative	negative	30%
59	Triple Negative	Ductal	86	4.5	3	IIIB	negative	negative	negative	10%
60	Triple Negative	Ductal	65	2.6	3	II2	negative	negative	negative	30%
61	Triple Negative	Ductal	73	6.5	3	IIIA	negative	negative	negative	20%
62	Triple Negative	Ductal	49	2.5	3	IIA	negative	negative	negative	70%
63	Triple Negative	Ductal	78	2.4	3	IIB	negative	negative	negative	40%
64	Metaplastic	undifferentiated sarcoma	63	1.5	3	IA	negative	negative	negative	50%
65	Metaplastic	chondroid-sarcomatous differentiation	84	3.5	3	IIA	negative	negative	negative	5%
66	Metaplastic	chondroid-sarcomatous differentiation	69	1.2	3	IA	negative	negative	negative	10%
67	Metaplastic	sarcomatous differentiation	47	9	3	IIIB	negative	negative	negative	50%
68	Metaplastic	sarcomatous differentiation	51	9	3	IIIB	negative	negative	negative	20%
69	Metaplastic	osteoblastic differentiation	73	6.5	3	IIIA	negative	negative	negative	20%
70	Metaplastic	chondroid-sarcomatous differentiation	78	2.4	3	IIB	negative	negative	negative	40%
