# Supplementary material for: MicroRNA-200 Family Modulation in Distinct Breast Cancer Phenotypes
Source: PLoS One. 2012 Oct 24;7(10):e47709. doi: 10.1371/journal.pone.0047709 (PMC3480416; doi:10.1371/journal.pone.0047709)
Supplement: Table S2 — Primers used for DNA methylation analysis. (PDF) [file pone.0047709.s007.pdf]

Table S2: Primers used for DNA methylation analysis

Promoter	Amplicon	Primer Sequence	Amplicon size
miR-200b-200a-429	1	Fwd: 5´-aggaagagagTGGGAGTTTAGGGGATATATTTG-3´<br>Re: 5´-cagtaatacgactcactatagggagaaggctACCCTAACACAAAAAATCAATTC-3´	240
	1 cells	Fwd: 5´-aggaagagagATGGGAGTTTAGGGGATATATTTGT-3´<br>Re: 5´-cagtaatacgactcactatagggagaaggctACTCTACCTCAACCAAAATCAAACC-3´	329
	2	Fwd: 5´-aggaagagagGATAGGTGTGTTTTTTGGGTTTTTA-3´<br>Re: 5´-cagtaatacgactcactatagggagaaggctACTACCCCTACCCCTACCCATATC-3´	200
miR-200c-141	1	Fwd: 5´-aggaagagagGAGTTTTTGGGGATATTTTTTGGT-3´<br>Re: 5´-cagtaatacgactcactatagggagaaggctAAAAATCCTAAACTAAACTTAAAACTACAA-3´	237
	2	Fwd: 5´-aggaagagagATGTTGTTGATATAGGGATAGGGGT-3´<br>Re: 5´-cagtaatacgactcactatagggagaaggctAACAAAACTCACCAAAAAATATCCC-3´	222
